# Genome Sequence of the Novel Reassortant Mammalian Orthoreovirus Strain MRV00304/13, Isolated from a Calf with Diarrhea from the United States

**DOI:** 10.1128/genomeA.00451-14

**Published:** 2014-05-29

**Authors:** Srivishnupriya Anbalagan, Tina Spaans, Ben M. Hause

**Affiliations:** Newport Laboratories Inc., Worthington, Minnesota, USA

## Abstract

Mammalian orthoreovirus (MRV) strain MRV00304/13 was isolated from diarrheic calves. The serotype-specific antigen σ1 was found to be 95% identical to that of bovine MRV1. All predicted viral proteins had >92% identity to those of MRV except µ2 and σ1s (80 and 72% identities, respectively), suggesting that MRV00304/13 is a novel reassortant MRV1.

## GENOME ANNOUNCEMENT

Mammalian orthoreovirus (MRV) is a species in the genus *Orthoreovirus*, family *Reoviridae*. MRVs are ubiquitous mammalian pathogens, infecting nearly all species (1). MRVs possess a 10-segment double-stranded RNA genome that enables reassortment and viral evolution (2, 3). There are three serotypes of MRV, which were determined classically using neutralizing antisera that are further supported by phylogenetic analyses of the antigenic determinant σ1 (3–5). Clinically, MRV infection ranges from asymptomatic to upper respiratory tract illness or enteritis and diarrhea (1).

Fecal samples were collected from 10-week-old bovine calves with diarrhea in Indiana in January 2014. Bacterial cultures failed to identify any significant organisms. Real-time reverse transcription-PCR for common bovine enteric viruses (groups A, B, and C rotavirus and bovine coronavirus) were weakly positive for group C rotavirus (threshold cycle [*C*_*T*_], 33.8). Virus isolation was attempted on rhesus monkey kidney (Marc145) cells, and cytopathic effects were observed on day 2. RNA sequencing was performed using an Ion Torrent Personal Genome Machine using a previously described methodology (6). Sequence assembly was conducted *de novo* using the DNAStar Lasergene 11 Core Suite. BLAST analysis of the assembled contigs identified all 10 segments of the genome.

The coding regions of the three large segments, L1, L2, and L3, were 3,804, 3,870, and 3,828 nucleotides (nt) in length, respectively, and had predicted proteins that were 98% identical to those of MRV. Likewise, the medium segments M2 (2,127 nt) and M3 (2,166 nt) coded for predicted proteins with high homologies (>92% identity) to those of MRV. In contrast, the M1 segment contained a 2,211-nt open reading frame with only 74% identity to MRV. The M1 gene codes for the µ2 protein, which was previously shown to be a growth determinant in cultured bovine aortic endothelial cells (7). The remaining four segments, S1 to S4, encoded 1,416-, 1,257-, 1,101-, and 1,098-nt open reading frames (ORFs) with 95, 99, 99, and 96% identities to MRV, respectively. The bicistronic S1 segment codes for σ1 and uses an overlapping ORF to code for the σ1s protein involved in viral spread *in vivo* and cell cycle arrest (8, 9). Protein σ1 was found to be 95% identical to a similar MRV1 isolate from a bovine animal from Maryland in 1959 (10). Interestingly, the σ1s protein of MRV00304/13 had only 72% identity to those of other MRVs.

Further work is necessary to determine the pathogenic potential of this novel reassortant bovine MRV.

### Nucleotide sequence accession numbers.

The complete genome sequence of MRV1 strain MRV00304/13 has been deposited in GenBank under accession no. KJ676379 (L1), KJ676380 (L2), KJ676381 (L3), KJ676382 (M1), KJ676383 (M2), KJ676384 (M3), KJ676385 (S1), KJ676386 (S2), KJ676387 (S3), and KJ676388 (S4).
